# Artificial Intelligence and Anticancer Drug Development—Keep a Cool Head

**DOI:** 10.3390/pharmaceutics16020211

**Published:** 2024-01-31

**Authors:** Caroline Bailleux, Jocelyn Gal, Emmanuel Chamorey, Baharia Mograbi, Gérard Milano

**Affiliations:** 1 Centre Antoine Lacassagne, Oncology Departement Unit, University Côte d’Azur, 06000 Nice, France; caroline.bailleux@nice.unicancer.fr; 2 Centre Antoine Lacassagne, Epidemiology and Biostatistics Department, University Côte d’Azur, 06000 Nice, France; jocelyn.gal@nice.unicancer.fr (J.G.); emmanuel.chamorey@nice.unicancer.fr (E.C.); 3 IRCAN Group, University Côte d’Azur, 06000 Nice, France; baharia.mograbi@unice.fr; 4 Centre Antoine Lacassagne, University Côte d’Azur, 33 Avenue de Valombrose, 06189 Nice, France

**Keywords:** serendipity, new drug discovery, oncology, artificial intelligence

## Abstract

Artificial intelligence (AI) is progressively spreading through the world of health, particularly in the field of oncology. AI offers new, exciting perspectives in drug development as toxicity and efficacy can be predicted from computer-designed active molecular structures. AI-based in silico clinical trials are still at their inception in oncology but their wider use is eagerly awaited as they should markedly reduce durations and costs. Health authorities cannot neglect this new paradigm in drug development and should take the requisite measures to include AI as a new pillar in conducting clinical research in oncology.

## 1. Introduction

**Figure 1:**
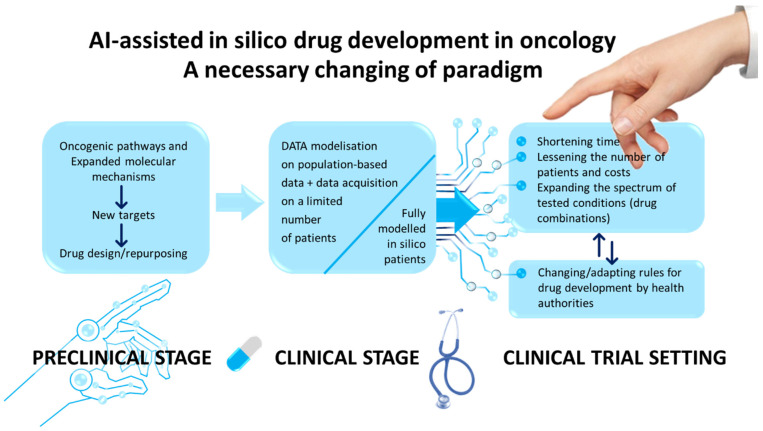
Different stages of AI-assisted in silico drug development.

Artificial intelligence (AI) is a general term comprising global machine learning and deep learning. One of the main applications of AI is to create mathematical models able to establish links between different types of information. In the medical field, AI can considerably accelerate discoveries as well as improve diagnoses and personalized treatments. The landscape of AI has recently evolved exponentially in the domain of cancer diagnosis and treatment [1]. In particular, AI now occupies a central place among challenges and potential solutions for improving the delivery of precision medicine in cancer [2]. More generally, numerous studies have pointed to the benefits of AI in drug discovery and cancer treatment [3,4]. A recent review on FDA-certified drugs in 2020 and 2021 indicated that 40% of the approved drugs were for various types of cancers [5]. This underlines the significant volume of scientific and medical work dedicated to the set-up of new anticancer drugs. This paper aims to objectively examine the main aspects of anticancer drug development where AI brings indisputable, significant advances. Anticancer drug development has generated a large quantity of AI-based reports and reviews; the main objective of the present opinion paper was not to perform an exhaustive synthesis of them. Rather, the present article aims to pinpoint the expected benefits as well as some limitations of AI in the area of new anticancer drug discovery and in the clinical development of them (Figure 1).

## 2. AI and Preclinical Anticancer Drug Development

AI can potentially radically transform clinical trial design and significantly impact preclinical drug development in several respects [6]. Among multiple advances conferred by AI, there is the possibility to more rigorously validate the hypotheses emerging from tumor profiling, define molecular mechanisms, and finally lead to innovative therapies. A review by Bhinder and coworkers [7] shed light on the spectrum of applications conferred by AI in oncology within the emerging domains of drug design and drug repurposing. Central to the applications conferred by AI in preclinical drug development are the domains of molecular screening and target identification thanks to the use of traditional machine learning and neutral networks. More precisely and regarding drug design, AI may generate in silico-designed molecules and analogs with given properties. AI has spawned a panel of potential new targets produced on the basis of CRISPR-based technology, offering a broad spectrum of targeted drugs with, interestingly, the anticipation of resistance mechanisms [8]. Protein structure prediction constitutes a complex domain of biology often requiring a computational approach. This area has been a challenge for scientists for many years. Broad potential for structure-based drug discovery springs from using AlphaFold2 (AF2), which is an AI-system that can predict 3D structures of proteins from amino acid sequences with atomic-level accuracy. A recent review by Yang Z et al. pointed to some limitations of AF2 prediction with particular relevance to the domain of anticancer therapy [9]. For instance, AF2 is unable to correctly predict proteins with multiple domains like transmembrane receptors, although it is clear that this type of target is a key component in the armamentarium of anticancer drugs. AF2 is not designed to predict the shape-changing of proteins in interaction with targeted drugs and AF2 performs poorly in predicting the effects of mutations on the protein structure. A positive point is the development of vector machine models that make it possible to predict pharmacokinetic properties, blood–brain barrier permeability, and intestinal absorption for anticancer drugs [7]. AI also offers the possibility to repurpose drugs beyond their existing medical indications, thus providing an original and economical alternative to conventional drug discovery [7].

However, we must keep in mind that there is historical evidence that many major drugs, in opposition to what AI can rationally generate, have been discovered fortuitously through random investigations of organisms; this is the so-called phenomenon of serendipity. As a historical illustration of serendipity and drug discovery, in 1962, there was the judicious observation that the presence of valproic acid, used as a solvent in cough syrups, provided a significant decrease in the number of seizures in epileptic patients, thus opening up an unanticipated window in the domain of anti-epileptic drugs [10]. Serendipity, thus, proves the role of chance in the identification of drugs of potentially great value in oncology. A typical example of what serendipity can offer is the unexpected discovery of cisplatin in the 1960s when a magnetic field produced by platinum electrodes was found to inhibit *E. coli* division [11,12]. This observation opened up a wide range of clinical applications covering cisplatin and its active analogs. As advocated by Louis Pasteur, chance favors the prepared mind. There may be some concerns that AI, by systematically assisting every step in drug development, could dampen some aspects of our scientific awareness and, thus, scale back certain individual abilities to maintain a prepared mind able to grasp an unexpected spurt of serendipity. Hence, as advocated by Lavazza et al., there is currently the risk that human creativity, imagination, and even divergence from AI logic might be discouraged [13].

## 3. AI and Clinical Trial Development

AI has the potential to improve the setting of clinical trials in several respects. First, the considerable progress offered by AI in cancer imaging and diagnosis should automatically produce a positive echo in clinical trial development [14]. Another advantage that AI has in clinical research is the simplification and speed of patient recruitment [15], particularly with the recent introduction of Chatbots like GPT-4 [16]. A systematic review and meta-analyses (50,000 patients and 19 data sets) have reported on the use of AI for cancer clinical trial enrollment [17], confirming the potential capacities of AI, whose performance in clinical trial enrollment is comparable, if not superior, to manual screening [15]. Exploiting big data and utilizing AI may further accelerate knowledge acquisition. Indeed, in oncology, clinical trials have markedly evolved, shifting from tumor-type-centered approaches to molecular classification and histology-diagnostic trials, with innovative, new clinical trial designs, such as umbrella or basket trials, and personalized combination treatment strategies tailored to individual biomarker profiles. AI should facilitate multiple data for their acquisition and treatment. However, it must be kept in mind that prospective clinical validation of AI-generated algorithms is necessary to ensure that the true improvement (including automated imaging and molecular data treatment) generated by AI holds up under various distribution shifts [18].

In cancer treatment, there is currently great enthusiasm for the development and clinical applications of cancer immunotherapy. This may, however, constitute a potential problem. With immunotherapy by checkpoint immunological inhibition, the difficulties lie mainly in trial design redundancy, leading to a ‘Wild West’ in drug development that features a complex interaction between commercial sponsors, clinical trials, and redundant development plans [19]. This problem is more widely encountered when considering the development of kinases inhibitors for the main oncogenic pathways [18]. There is, in this context, a true sprouting of the so-called me-too drugs where the benefit for the patient versus the one for drug companies is often hard to distinguish for the scientific, therapeutic eye. It is clear that AI should be able to markedly improve this situation and bring answers and innovative solutions to this specific context. For instance, as previously underlined by our group [20], the setting up of in silico trials could be an interesting option. In silico clinical trials take advantage of the modelisation of cumulated clinical experience and biological data pertaining to previously developed compounds that belong to the same category of drugs. Treatment design can also benefit from an in silico development strategy. For instance, Bajard et al. [21] compared seven experimental designs for randomized clinical trials using in silico simulations. The objective of the study was to show that in silico simulation could assist in the selection of the experimental design for a future clinical trial in terms of power and accuracy of the estimation of treatment effects. It is important to note that the conventional rules for drug development are becoming inadequate considering the limited number of patients available for therapeutic trials in the field of immunotherapy; for instance, as there is a wide range of potentially active schedules to test with, in particular, multiple combinations to take into consideration. In this context, there is a real risk in a conventional therapeutic trial setting of missing an opportunity to highlight a particularly active drug combination because of the limited number of patients. In this respect, compared to conventional trial design and conduct, in silico trials would incorporate far fewer but more informative patients [22] and could be carried out more quickly, at a lower cost. In short, the general idea behind in silico trials is to project patient responses and outcomes by capturing individual critical parameters from a limited number of patients and incorporating this information into an AI-designed model built on cumulated anterior data gained using similar drugs. Compared to traditional models, AI-designed models, mainly based on machine learning and deep-learning algorithms, confer advantages of great flexibility in relation to the volume of data that can be analyzed. They are also disadvantages such as overfitting or underfitting. In some cases, these models may also be too complex for a clear interpretation. There is also the possible problem of unbalanced data whose proportions by class are uncertain. Globally, this promising, achievable strategy calls for changes on the part of health authorities in the currently strict, more or less rigid, criteria for clinical validation. In general terms, AI could also make it possible to avoid duplicate development programs as duplication may erode the resources necessary for true innovation, which constitutes one of the main motivations for patients to participate and for clinicians to accept clinical trial proposals [19]. Finally, it appears essential that those involved in clinical research, whether in industry or academia, are conscious of this unavoidable methodological evolution based on AI and incorporate it in future strategies for anticancer drug development.

So far, we have considered AI-assisted in silico clinical trials from the angle of including a limited number of patients in a trial that incorporates cumulated, modelised knowledge gained from a large set of patients previously treated in similar conditions. Another complementary approach to in silico clinical trials consists of recruiting totally simulated patients and applying given changes in representative parameters such as biological constants or tumor characteristics. In such a setting, a report was published on the Novadiscovery’s jinkō trial simulation platform [15] which was used to predict AstraZeneca’s phase III clinical trial FLAURA2 [23]. It is fascinating to note that the simulation took just one month to set up, whereas the clinical trial lasted 3 years. In fact, the platform was able to predict that adding chemotherapy to osimertinib in patients with EGFR-mutated, non-small cell lung cancer significantly increased progression-free survival. An alternative to simulated patients can be envisaged using big data. By aggregating and structuring computerized data from patients’ medical records [24,25] and using artificial intelligence algorithms, it is possible to automatically identify patients eligible for clinical trials and, thus, create ‘real simulated patients’. This will reduce the duration and cost of therapeutic trials.

Altogether, there are strong arguments in favor of in silico clinical trial development in oncology. It, thus, appears necessary for health authorities to thoroughly revise the rules for setting up clinical trials, incorporating AI and in silico methodology [26]. These rules will have to include the notion of result reproducibility if they are to be accepted by the medical and scientific community [27].

## 4. Perspectives and Conclusions

This position paper highlights the growing place of AI in anticancer drug development, as it is clear that AI will take over drug design and clinical trial settings. We have pointed out some of the limitations of AI, such as the unavoidable loss of serendipity as a source of drug discovery and the relative lack of concrete, pertinent applications for AI-designed in silico clinical trials when costs for therapeutic innovation in cancer are markedly increasing [28]. This view concurs with recent recommendations made by the FDA [29] and the EMEA [30,31] on using AI in the entire lifecycle of medicines from drug discovery to the post-authorization setting. The EMEA strategy for AI involves several keys points including the need to leverage digital technology and AI assistance in decision making. This renders digital innovation capable of exploring, piloting, and developing solutions and processes across the drug regulation spectrum with the ultimate goal of engaging with diverse stakeholders like main regulators (FDA). There are other domains where the contribution and performance of AI are particularly eagerly awaited, like emerging technologies and more accurate representation of patients’ disease stage, including patient-derived organoids enabling rapid drug-response evaluation [6]. Finally, as Wang and coworkers advocated [32], we still need to evaluate the accuracy and usefulness of AI-powered systems in cancer area. In this respect, a recent review by Kumar and coworkers underlines the importance of reliable AI systems, emphasizing the complexity of developed AI models which can possibly impact their interpretability [33]. This view is particularly true for accurately interpreting AI outputs in anticancer drug development, avoiding over-reliance on potentially flawed results with significant impacts on costs linked to wrong paths. With emerging big data, AI has the potential to enable innovative trials and faster development of new trials. Therefore, it offers the possibility, through the creation of ‘digital’ patients, of lowering risks to human health; this does not, however, spell the end of pharmaceutical trials on humans, which remain essential.

## References

[1] Subbiah V. (2023). The next generation of evidence-based medicine. Nat. Med..

[2] Schilsky R.L., Longo D.L. (2022). Closing the Gap in Cancer Genomic Testing. N. Engl. J. Med..

[3] Elkhader J., Elemento O. (2022). Artificial intelligence in oncology: From bench to clinic. Semin. Cancer Biol..

[4] Rani P., Dutta K., Kumar V. (2022). Artificial intelligence techniques for prediction of drug synergy in malignant diseases: Past, present, and future. Comput. Biol. Med..

[5] Passi I., Salwan S., Kumar B. (2023). US-FDA Approved Drugs in 2020 and 2021: A Review. Mini-Rev. Med. Chem..

[6] Gerstberger S., Jiang Q., Ganesh K. (2023). Metastasis. Cell.

[7] Bhinder B., Gilvary C., Madhukar N.S., Elemento O. (2021). Artificial Intelligence in Cancer Research and Precision Medicine. Cancer Discov..

[8] Wessels H.H., Stirn A., Mendez-Mancilla A., Kim E.J., Hart S.K., Knowles D.A., Sanjana N.E. (2023). Prediction of on-target and off-target activity of CRISPR-Cas13d guide RNAs using deep learning. Nat. Biotechnol..

[9] Yang Z., Zeng X., Zhao Y., Chen R. (2023). AlphaFold2 and its applications in the fields of biology and medicine. Signal Transduct. Target. Ther..

[10] Corrales-Hernandez M.G., Villarroel-Hagemann S.K., Mendoza-Rodelo I.E., Palacios-Sanchez L., Gaviria-Carrillo M., Buitrago-Ricaurte N., Espinosa-Lugo S., Calderon-Ospina C.A., Rodriguez-Quintana J.H. (2023). Development of Antiepileptic Drugs throughout History: From Serendipity to Artificial Intelligence. Biomedicines.

[11] Cheung-Ong K., Giaever G., Nislow C. (2013). DNA-damaging agents in cancer chemotherapy: Serendipity and chemical biology. Chem. Biol..

[12] Gibson D. (2021). Pt(IV) Anticancer Prodrugs—A Tale of Mice and Men. ChemMedChem.

[13] Lavazza A., Farina M. (2023). Infosphere, Datafication, and Decision-Making Processes in the AI Era. Topoi.

[14] Ladbury C., Amini A., Govindarajan A., Mambetsariev I., Raz D.J., Massarelli E., Williams T., Rodin A., Salgia R. (2023). Integration of artificial intelligence in lung cancer: Rise of the machine. Cell Rep. Med..

[15] Jinkō: A Complete Solution for Trial Simulation & Design Optimization. https://www.novadiscovery.com/jinko/.

[16] Haug C.J., Drazen J.M. (2023). Artificial Intelligence and Machine Learning in Clinical Medicine, 2023. N. Engl. J. Med..

[17] Chow R., Midroni J., Kaur J., Boldt G., Liu G., Eng L., Liu F.F., Haibe-Kains B., Lock M., Raman S. (2023). Use of artificial intelligence for cancer clinical trial enrollment: A systematic review and meta-analysis. J. Natl. Cancer Inst..

[18] Swanson K., Wu E., Zhang A., Alizadeh A.A., Zou J. (2023). From patterns to patients: Advances in clinical machine learning for cancer diagnosis, prognosis, and treatment. Cell.

[19] Beaver J.A., Pazdur R. (2022). The Wild West of Checkpoint Inhibitor Development. N. Engl. J. Med..

[20] Gal J., Milano G., Ferrero J.M., Saada-Bouzid E., Viotti J., Chabaud S., Gougis P., Le Tourneau C., Schiappa R., Paquet A. (2018). Optimizing drug development in oncology by clinical trial simulation: Why and how?. Brief. Bioinform..

[21] Bajard A., Chabaud S., Cornu C., Castellan A.C., Malik S., Kurbatova P., Volpert V., Eymard N., Kassai B., Nony P. (2016). An in silico approach helped to identify the best experimental design, population, and outcome for future randomized clinical trials. J. Clin. Epidemiol..

[22] Boissel J.P., Perol D., Decousus H., Klingmann I., Hommel M. (2021). Using numerical modeling and simulation to assess the ethical burden in clinical trials and how it relates to the proportion of responders in a trial sample. PLoS ONE.

[23] (2023). EGFR-Mutant NSCLC: Chemo-TKI Bests TKI Alone. Cancer Discov..

[24] Schiappa R., Contu S., Culie D., Chateau Y., Gal J., Pace-Loscos T., Bailleux C., Haudebourg J., Ferrero J.M., Barranger E. (2023). Validation of RUBY for Breast Cancer Knowledge Extraction From a Large French Electronic Medical Record System. JCO Clin. Cancer Inform..

[25] Schiappa R., Contu S., Culie D., Thamphya B., Chateau Y., Gal J., Bailleux C., Haudebourg J., Ferrero J.M., Barranger E. (2022). RUBY: Natural Language Processing of French Electronic Medical Records for Breast Cancer Research. JCO Clin. Cancer Inform..

[26] Musuamba F.T., Skottheim Rusten I., Lesage R., Russo G., Bursi R., Emili L., Wangorsch G., Manolis E., Karlsson K.E., Kulesza A. (2021). Scientific and regulatory evaluation of mechanistic in silico drug and disease models in drug development: Building model credibility. CPT Pharmacomet. Syst. Pharmacol..

[27] Karr J., Malik-Sheriff R.S., Osborne J., Gonzalez-Parra G., Forgoston E., Bowness R., Liu Y., Thompson R., Garira W., Barhak J. (2022). Model Integration in Computational Biology: The Role of Reproducibility, Credibility and Utility. Front. Syst. Biol..

[28] Tannock I.F., Bouche G., Goldstein D.A., Goto Y., Lichter A.S., Prabhash K., Ranganathan P., Saltz L.B., Sonke G.S., Strohbehn G.W. (2023). Patient-centered, self-funding dose optimization trials as a route to reduce toxicity, lower cost, and improve access to cancer therapy. Ann. Oncol..

[29] Drug F. (2022). Modeling & Simulation at FDA. https://www.fda.gov/science-research/about-science-research-fda/modeling-simulation-fda.

[30] Agency E.M. (2020). Advancing Regulatory Science in the EU—New Strategy Adopted. https://www.ema.europa.eu/en/news/advancing-regulatory-science-eu-new-strategy-adopted.

[31] Musuamba F.T., Bursi R., Manolis E., Karlsson K., Kulesza A., Courcelles E., Boissel J.P., Lesage R., Crozatier C., Voisin E.M. (2020). Verifying and Validating Quantitative Systems Pharmacology and In Silico Models in Drug Development: Current Needs, Gaps, and Challenges. CPT Pharmacomet. Syst. Pharmacol..

[32] Wang H., Fu T., Du Y., Gao W., Huang K., Liu Z., Chandak P., Liu S., Van Katwyk P., Deac A. (2023). Scientific discovery in the age of artificial intelligence. Nature.

[33] Kumar K.S., Miskovic V., Blasiak A., Sundar R., Pedrocchi A.L.G., Pearson A.T., Prelaj A., Ho D. (2023). Artificial Intelligence in Clinical Oncology: From Data to Digital Pathology and Treatment. Am. Soc. Clin. Oncol. Educ. Book.

